# Emotional Labour and Work-Family Conflict in Voice-to-Voice and Face-to-Face Customer Relations: A Multi-Group Study in Service Workers

**DOI:** 10.5964/ejop.v16i4.1838

**Published:** 2020-11-27

**Authors:** Federica Emanuel, Lara Colombo, Stefania Santoro, Claudio G. Cortese, Chiara Ghislieri

**Affiliations:** a Department of Philosophy and Education Sciences, University of Turin, Turin, Italy; b Department of Psychology, University of Turin, Turin, Italy; University of Wollongong, Wollongong, Australia

**Keywords:** emotional dissonance, customer verbal aggression, service work, work-family conflict

## Abstract

Professions that involve interaction with customers entail great emotional effort: workers are required to show emotions different from their true feeling and they experienced emotional dissonance and verbal aggression from customers. These job demands can generate discomfort and the effects of emotional labour can “expand” in other life domains. The study investigated the relationship among emotional dissonance, customer verbal aggression, affective discomfort at work and work-family conflict, considering differences between two groups of service workers: call centre agents (CA; N = 507, voice-to-voice relation with customers) and supermarket cashiers (SC; N = 444, face-to-face relation with customers). Results showed that emotional dissonance and customer verbal aggression had a positive relationship with work-family conflict, the mediational role of affective discomfort emerged in both groups; different effects of job demands in subsamples appeared. Suggestions for organisations and work processes emerged in order to identify practical implications useful to support employees in coping with emotional labour and to promote well-being and work-family balance.

Recent changes in the labour market and the rapid development of service companies have shown how the emotional aspect of work is significant not only as an outcome but also as a job demand (Hülsheger & Schewe, 2011; Schaufeli & Taris, 2014). The professions that involved interaction and/or direct contact with customers demanded working on tasks and spending mental and physical effort, also required emotional labour and managing emotions and relations with customers (Zapf, 2002). These characteristics of person-oriented professions can generate feelings of discomfort and the effects of emotional labour can “expand” negatively in other life domains (Zapf, 2002). The relationship between emotional labour and work-family conflict (WFC) is still little investigated (Cheung & Tang, 2009; Kinman, 2009; Montgomery, Panagopolou, & Benos, 2005; Montgomery, Panagopolou, De Wildt, & Meenks, 2006; Yanchus, Eby, Lance, & Drollinger, 2010) and in Italy studies on this topic are scant (Emanuel, Molino, & Ghislieri, 2014b; Ghislieri, Ricotta, & Colombo, 2012).

Among the theoretical models able to understand several aspects affecting well-being at work, the job demands-resources theory (JD-R theory; Bakker & Demerouti, 2017) has received much consideration by scholars. The theory assumes that well-being is affected by two categories of aspects, job demands and job resources: job demands are mainly responsible for health degradation processes, job resources are mainly responsible for motivational processes. This theory is flexible and adaptable to different work environments and jobs, identifying specific job demands and job resources. Job demands are “physical, psychological, social, or organizational aspects of the job that require sustained physical and/or psychological (cognitive and emotional) effort or skills and are therefore associated with certain physiological and/or psychological costs” (Bakker & Demerouti, 2007, p. 312). Job demands are not negative by definition; they become stressors when meeting those demands requires a high effort that the person cannot adequately cope with. Job resources represent the second set of job characteristics and “refer to those physical, psychological, social, or organizational aspects of the job that are either/or: functional in achieving work goals; reduce job demands and the associated physiological and psychological costs; stimulate personal growth, learning, and development” (Bakker & Demerouti, 2007, p. 312). High levels of job-related stressors and a lack of job resources may negatively affect employees’ well-being (Bakker & Demerouti, 2007, 2017). Starting to these considerations, this study aims to investigate the relationship among job demands typical for service work, affective discomfort at work and WFC in two different jobs: call centre agents, characterised by a voice-to-voice relation with customers, and supermarket cashiers, characterised by a face-to-face relation with customers. The labour market for call centre agents and supermarket cashiers is characteristic of a “secondary labour market” of insecure, poorly paid jobs without any career opportunities. Traditionally, these service work occupations were once considered “transitory” and appropriate for low skills. Currently in Italy, a country characterised by a weak labour market, high job insecurity and unemployment, call centre and supermarket jobs have become occupations in which personnel stop working for long periods of time. These occupations are no longer characterised by high turnover because people cannot change: intense changes in the labour market have made it more complex for individuals to adapt to work and make occupational choices (Savickas et al., 2009).

## Emotional Labour in Service Work

In customer service settings, providing “service with a smile” (Grandey, Fisk, Mattila, Jansen, & Sideman, 2005) is a job requirement used to promote the customer’s intention to return and their satisfaction. Organisations have clear expectations about the interaction between their employees and customers. For example, a frequent organisational goal is customer satisfaction, which is reached through employees’ customer orientation that can be displayed by employees being very polite, friendly or asking about the customer’s needs (Diefendorff, Richard, & Croyle, 2006; Grandey, 2000; Zapf, 2002). Specifically, employees are required to display emotions that are in line with organisational expectations of what emotional displays are appropriate or expected in certain situations (Grandey, 2000; Hochschild, 1983). Emotional display rules have been explored in a wide array of occupational contexts (Hülsheger & Schewe, 2011; Kenworthy, Fay, Frame, & Petree, 2014): the typical model is that positive emotions should be expressed and negative emotions suppressed.

Emotional labour is a fundamental component of service work (Kinman, 2009). In service work interactions with clients are usually repetitive and scripted and an excessive level of emotional control may be required to preserve positive interactions (Kinman, 2009; Zapf, 2002; Zapf, Vogt, Seifert, Mertini, & Isic, 1999). Moreover, is important that workers maintain a warm and friendly interaction with customers even when they undergo abuse or verbal aggression (Grandey et al., 2004; Zapf, Isic, Bechtoldt, & Blau, 2003).

Hochschild (1983) affirms that jobs involving extensive interpersonal contact with customers or clients necessarily entail emotional labour. Moreover, jobs that require emotional labour have three characteristics (Hochschild, 1983): (1) they require face-to-face or voice-to-voice contact with the public; (2) they require worker to produce an emotional state in the customer or client; and (3) they allow the employer, through training and supervision, to exert some control over the emotional activities of employees. Emotional labour can be defined as the process of regulating feelings and expressions as part of the work role (Grandey, 2000; Hülsheger & Schewe, 2011; Zapf et al., 1999). In fact, emotions play an important role in employee/customer relations. Companies and managers highlight the importance of this relationship and employees are encouraged formally (or informally) by their organisations to display emotions during face-to-face or voice-to-voice interactions that conform to specific display rules defined by the specific organisation (Grandey, 2000; Grandey & Gabriel, 2015; Hülsheger & Schewe, 2011; Zapf et al., 1999).

The present study involves employees of two different service contexts: call centre agents and supermarket cashiers.

Some scholars have defined call centre jobs as an advanced form of Taylorism (Molino et al., 2016; Zapf et al., 2003) mainly for the labour division, simplification of tasks, standardisation and time pressure (Bélanger & Edwards, 2013). Moreover, call centre jobs involve the voice-to-voice interaction with customers and high emotional effort is required for customer relations and for the suppression of negative emotions caused by unfriendly or angry customers (Dormann & Zapf, 2004; Grandey et al., 2004; Zapf et al., 2003). Literature highlight that emotional dissonance is the principal strain phenomenon in call centre work (Emanuel et al., 2014a; Ghislieri et al., 2012; Molino et al., 2016; Zapf et al., 2003).

Studies concerning supermarket cashiers are few; the majority of studies analyse physical distress and illness since supermarket workers are known to be an at-risk population for musculoskeletal disorders (Forcier et al., 2008; Sansone, Bonora, Boria, & Meroni, 2014). Rafaeli (1989), in his pioneering study of supermarket workers in Israel, identified that the work organisation is characterised by rigid discipline and control, which can be seen in the special distribution of cashiers and the control of their body and their emotions. Moreover, the cashier’s job involves more face-to-face interaction and physical proximity to the customer than any other employment. Supermarket cashiers are still expected to suppress their feelings in front of offensive customers and display emotions desirable for the organisation, such as friendliness. The cashier/customer experience can range from pleasant to threatening: customers are not only a source of satisfaction, incentive and social interaction, but also a source of violence and suffering for supermarket cashiers (Rafaeli, 1989).

This study considers emotional dissonance and customer verbal aggression as job demands (Grandey, 2000; Schaufeli & Taris, 2014), typical of service employment. Emotional dissonance arises when employees’ expressed emotions, that are considered acceptable by the organisation, do not represent their true feelings (Grandey & Gabriel, 2015; Hülsheger & Schewe, 2011; Rafaeli & Sutton, 1987; Zapf, 2002). Service occupations are considered emotionally demanding for workers because they have to express certain emotions that may not be felt or even be opposed to those internally perceived in the situation. During interactions in person (face-to-face) or mediated by telephone (voice-to-voice), workers express emotions required by the organisation and meet customer expectations, although relations are not always easy to manage (Kinman, 2009). However, several scholars underline that emotional dissonance may be stressful and detrimental to health: a positive association emerged with psychological strain, emotional exhaustion, psychosomatic complaints, WFC and lower job satisfaction (e.g., Emanuel, Colombo, & Ghislieri, 2014a; Grandey & Gabriel, 2015; Hülsheger & Schewe, 2011; Kenworthy et al., 2014; Molino et al., 2016; Zapf et al., 1999).

Another job demand considered in this study, typical of service work, is customer verbal aggression that refers to aggression from unfriendly, rude and/or unsatisfied customers, shown through shouting at service workers and using negative verbal expressions (e.g., yelling, insulting, and cursing; Dormann & Zapf, 2004; Grandey et al., 2004; Grandey et al., 2005). In the European representative sample (Eurofound, 2012), 11% of workers reported that they experienced verbal abuse in the previous month and 2% say they were exposed to physical violence in the last year: exposure to aggression from leaders, co-workers, subordinates or customers was assumed to have profound negative consequences for the health and well-being of the target employed. Thus, customer verbal aggression might be particularly stressful for the employee and problematic for the organisation (Dormann & Zapf, 2004; Grandey et al., 2004). Moreover, aggressive customers express and, in turn, foster emotions in employees that they could not show according to emotional rules in service organisations (Grandey et al., 2004; Molino et al., 2016).

Considering these characteristics of service work, we hypothesize that:

Hypothesis 1: (a) emotional dissonance is positively associated with affective discomfort at work; and (b) customer verbal aggression is positively associated with affective discomfort at work.

## Work-Family Conflict, Strain, and Emotional Labour

Traditionally, the work-family literature grounded in role theory (Kahn, Wolfe, Quinn, Snoek, & Rosenthal, 1964), which assumes that multiple roles lead to role stress that, in turn, results in strain. WFC has been defined as “a form of inter-role conflict in which the role pressures from the work and family domains are mutually incompatible in some respect. That is, participation in the work (family) role is made more difficult by virtue of participation in the family (work) role” (Greenhaus & Beutell, 1985, p. 77).

Researchers have considered a number of different variables as possible antecedents of WFC (Amstad, Meier, Fasel, Elfering, & Semmer, 2011; Ghislieri & Colombo, 2014; Nohe, Meier, Sonntag, & Michel, 2015): role and job demands are the main determinants of WFC. Given the growing concern about WFC, much attention has been directed toward the potential outcomes of such conflict; for example, low levels of job satisfaction, lack of organisational commitment, absenteeism and turnover (Amstad et al., 2011; Ford, Heinen, & Langkamer, 2007; Ghislieri & Colombo, 2014; Nohe et al., 2015). Moreover, studies report a significant relation with burnout and physical and psychological stress-related outcomes (Frone, Russell, & Cooper, 1997; Kinnunen, Geurts, & Mauno, 2004).

Some researchers suggest that strain is likely to affect the perception and experience of WFC. If an individual suffers from psychological job-related strain, he or she will consequently have fewer resources to cope with work and family responsibilities and demands, as highlighted in the job demands–resources theory (Bakker & Demerouti, 2017). The JD–R theory draws on Conservation of resource theory and particularly personal resource theory (Hobfoll, 2001, 2002) to explain an erosion process through which job demands deplete personal energy resources, leading to emotional exhaustion (Bakker, Demerouti, & Dollard, 2008). This consideration is consistent with the view that resources are limited and may eventually diminish because of changes in well-being and health (Edwards & Rothbard, 2000). In fact, several studies indicate the possibility of WFC as an outcome of the negative effects of emotional exhaustion or strain. For example, Kelloway, Gottlieb, and Barham (1999) proposed that distress is likely to affect the perceived frequency and intensity of difficulties of combining work and family roles. Similarly, stressful work conditions may lead to more WFC (Ford et al., 2007) as well as burnout (Westman, Etzion, & Gortler, 2004), job dissatisfaction (Britt & Dawson, 2005), psychological and physical stress symptoms (Kinnunen et al., 2004; Steinmetz, Frese, & Schmidt, 2008).

This study examines the relationship between emotional dissonance and customer verbal aggression with affective discomfort at work. Affective discomfort is an indicator of strain, which referred to the intensity of emotions experienced at work: specifically, high levels of negative emotions are associated with low levels of well-being (Biggio & Cortese, 2013; Emanuel et al., 2014a; Molino et al., 2016; Van Katwyk, Fox, Spector, & Kelloway, 2000; Warr, 1990). According to several studies (Grandey, Tam, & Brauburger, 2002; Weiss & Cropanzano, 1996), events that occur at work, depending on the work environment and the disposition of the individual, may result in emotional reactions; moreover, when these responses are aggregated over time, they can influence the overall feelings about the job experiences (Weiss & Cropanzano, 1996; Grandey, Tam, & Brauburger, 2002).

Though studies are scant, several have established a relationship between emotion labour and WFC (Carlson, Ferguson, Hunter, & Whitten, 2012; Cheung & Tang, 2009; Lawson, Davis, Crouter, & O’Neill, 2013; Montgomery et al., 2005; Sanz-Vergel, Rodríguez-Muñoz, Bakker, & Demerouti, 2012; Seery, Corrigall, & Harpel, 2008; Wagner, Barnes, & Scott, 2014; Yanchus et al., 2010). The emotion regulation performed in one role depletes energy and resources (Edwards & Rothbard, 2000), which motivates the person to conserve resources in the other role (Grandey & Krannitz, 2016; Hobfoll, 2001, 2002). Emotional labour in the workplace will produce negative emotions such as anxiety, anger or guilt (Judge, Ilies, & Scott, 2006) and emotion suppression elicits negative emotions that are carried to the other domain (Grandey & Krannitz, 2016; Wagner et al., 2014).

Thus, we assume that:

Hypothesis 2: (a) emotional dissonance is positively associated with WFC; and (b) customer verbal aggression is positively associated with WFC.

Hypothesis 3: affective discomfort at work is positively related to WFC, thus playing a mediating role between emotional dissonance and customer verbal aggression on the one hand, and WFC on the other.

**Figure 1:**
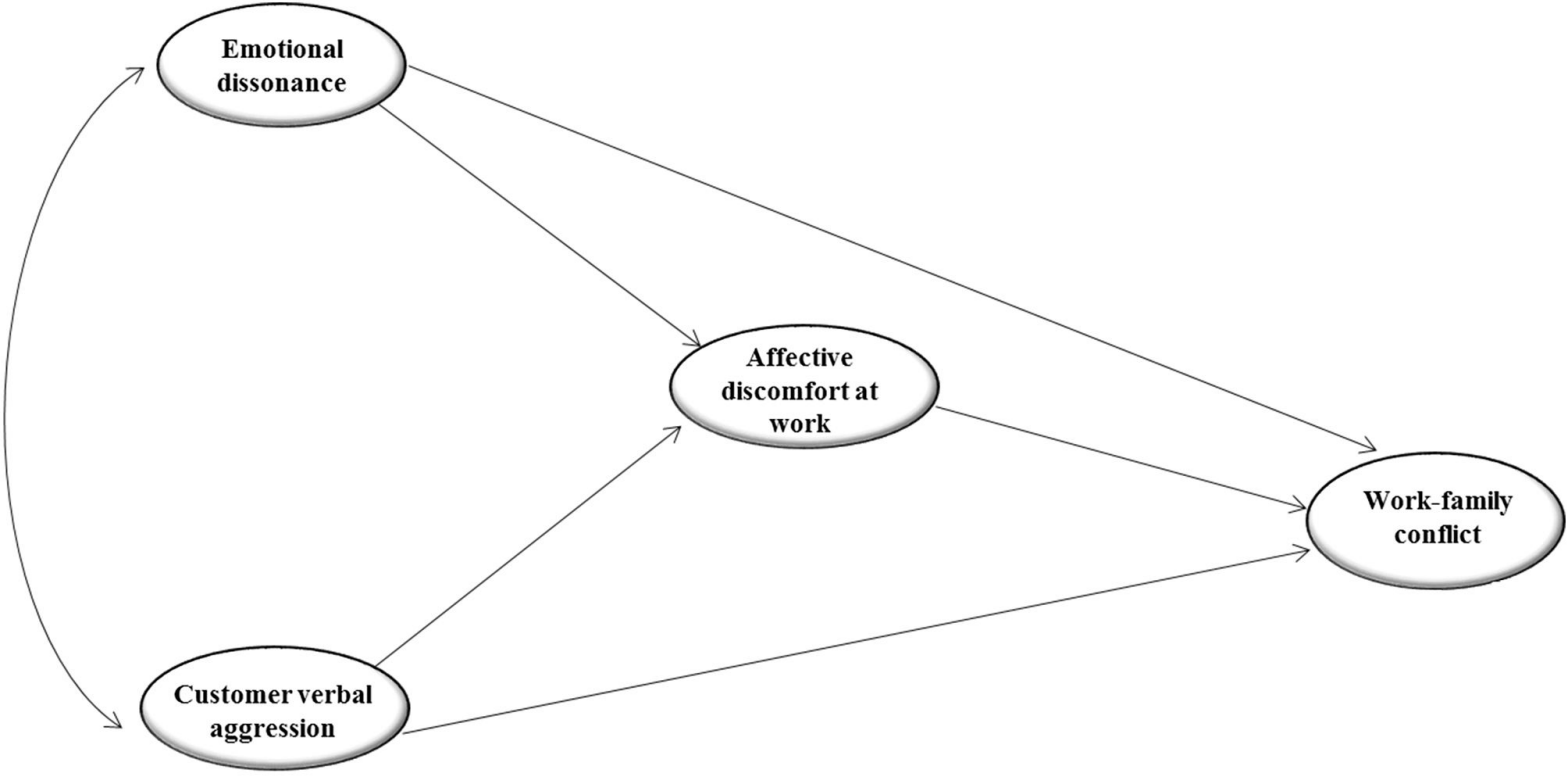
The theoretical model.

Figure 1 summarizes the hypothesized model in which two job demands, typical of service work (emotional dissonance and customer verbal aggression), are directly and indirectly, through the mediation of affective discomfort at work, related to WFC in service employees.

Since different job tasks and organisational contexts can generate different emotional and psychological dynamics (Kinman, 2009; Molino et al., 2016), the hypotheses are tested across the two types of service employees: the first group is call centre agents (CA), characterised by a voice-to-voice relation with customers; the second is supermarket cashiers (SC), characterised by a face-to-face relation with customers. The perspective from which we studied these possible differences is explorative; therefore, we do not define specific hypotheses with regard to this.

## Method

### Procedure and Participants

The research involved workers belonging to two organisational contexts of the North-West of Italy: a group of supermarket cashiers (71.96% of employees, *N* = 444) and a group of call centre agents (42.07% of employees, *N* = 507) fill out the self-report questionnaire. In both job environments, the aim of the study was explained to employees by sending an e-mail from management; moreover, a specific communication was published in the intranet. The first page of questionnaire showed the introduction and information about the study including the aim of the research and the informed consent. Moreover, anonymity, confidentiality of the data and the voluntary nature of participation in the study were emphasised and guaranteed.

The CA sample included 62.1% females and 37.9% males; their average age was 41.17 years (*SD* = 7.49; min = 21; max = 59). Among them, 24.9% were unmarried, 67.9% married or cohabiting, 7.3% separated, divorced or widowed and 59.8% had children. Most of the CA participants had finished high school (82.4%); 12.1% had a bachelor’s or master’s degree; and 5.3% had finished middle school. Most of the participants had a permanent contract (98.0%) and a full-time job (70.8%); mean organisational tenure was 16.74 years (*SD* = 7.88; min = 0; max = 37).

The SC sample included 100% females: this was representative of the gender distribution in organisation and of the occupation that, also in Italy, was predominantly performed by women. The SC average age was 34 (*SD* = 8.14; min = 19; max = 57). Among them, 33.7% were unmarried, 58.1% married or cohabiting, 8.2% separated, divorced or widowed; and 51.0% had children. Half of the SC participants had finished high school (58.2%), 9.2% had a bachelor’s or master’s degree; and 32.6% had finished middle school. Half of the participants had a permanent contract (56.7%) and a part-time job (84.4%); mean organisational tenure was 5.28 years (*SD* = 3.74; min = 0; max = 18).

### Measures

WFC was assessed with 5 items developed by Colombo and Ghislieri (2008). All items were scored on a 7-point scale, ranging from 1 = *never* to 7 = *all of the time*. An example item is “The amount of time my job takes up makes it difficult to fulfil family responsibilities”. Cronbach’s alpha for this study was .88 (.89 for CA, .87 for SC).

Affective discomfort at work was assessed with 6 items on Warr’s (1990) scale. All items were scored on a 6-point scale, ranging from 1 = *never* to 6 = *all of the time*. Respondents are asked, thinking of the past few weeks, how much of the time their job had made them feel, for example, “depressed” or “gloomy”. Cronbach’s alpha was .85 (.86 for CA, .84 for SC).

Emotional dissonance was assessed with 4 items developed by Zapf et al. (1999). All items were scored on a 6-point scale, ranging from 1 = *never* to 6 = *always*. Respondents are asked, for example, how often during their work, they have to “Display emotions which do not correspond to inner feelings”. Cronbach’s alpha was .84 (.93 for CA, .77 for SC).

Customer verbal aggression was assessed with 4 items by Dormann and Zapf (2004). All items were scored on a 6-point scale, ranging from 1 = *strongly disagree* to 6 = *strongly agree*. An example item is “Customers personally attack us verbally”. Cronbach’s alpha was .81 (.87 for CA, .79 for SC).

### Data Analysis

Data analyses were performed using IBM SPSS Statistics (Version 24): descriptive statistics (mean and standard deviation) and α reliabilities (Cronbach’s α) were carried out for each scale in both groups separately (CA and SC). Pearson bivariate correlations (*r*) were used to examine the interrelationships between variables, the differences in the means of variables between CA and SC were examined by using the analysis of variance (*t*-test for independent samples).

Multi-group structural equation model (SEM) was performed using Mplus (Version 7) in order to assess differences across groups in the hypothesised model. The method of estimation was maximum likelihood (ML) and hypotheses are specified a priori leading to the choice of a partial mediation model. According to literature (Bentler, 1990; Bollen & Long, 1993), the model was evaluated by several goodness-of-fit criteria: the χ^2^ goodness-of-fit statistic; the root mean square error of approximation (RMSEA); the comparative fit index (CFI); the Tucker Lewis index (TLI); and the standardised root mean square residual (SRMR). To construct latent variables, a parcelling method was used: the indicators of the latent variables are parcelled (aggregate-level indicators comprising an average of two or more items) for each latent variable. Studies report that using parcels as indicators of a construct is better than using more items since it reduces type I errors in the item correlations and lessens the likelihood of a priori model misspecification (Bandalos, 2002; Little, Cunningham, Shahar, & Widman, 2002; Yang, Nay, & Hoyle, 2010). All parcels presented significant loadings in the structural equation model calculated.

To address the common method variance issue, we performed the Harman’s single-factor test (Podsakoff, MacKenzie, Lee, & Podsakoff, 2003) using confirmatory factor analysis. Results indicated that one single factor could not account for the variance in the data, since all measures of goodness-of-fit indicated that the model did not fit the data, χ^2^(20) = 1473.34, *p* < .01, RMSEA = .28, 90% CI [.26, .29], CFI = .53, TLI = .34, SRMR = .12; therefore, the threat of common method bias was unlikely.

## Results

Table 1 shows the means, standard deviations, bivariate correlations among the study variables and internal consistency of each scale, separately for the CA and SC samples. All α values met the criterion of .70 (Nunnally & Bernstein, 1994) as they ranged between .77 and .93.

**Table 1:** Item Means, Item Standard Deviation, Cronbach’s Alphas and Correlations Among the Study Variables for CA (N = 507) and SC (N = 444)

Study variable	CA		SC		1	2	3	4
	*M*	*SD*	*M*	*SD*				
1. WFC	3.25	1.28	2.80	1.12	.89/.87	.43**	.39**	.21**
2. Affective discomfort at work	3.08	1.13	2.88	1.03	.41**	.86/.84	.43**	.27**
3. Emotional dissonance	3.97	1.28	3.71	1.11	.20**	.20**	.93/.77	.29**
4. Customer verbal aggression	4.44	1.12	3.85	1.15	.33**	.21**	.28**	.87/.79

*Note.* Correlations for the CA above the diagonal; correlations for the SC below the diagonal. Cronbach’s α for CA/SC sample on the diagonal. CA = Call centre agents; SC = Supermarket cashiers; WFC = Work-family conflict.

*p < .05. **p < .01.

All the significant correlations between the variables were in the expected directions. WFC and affective discomfort were positively correlated with job demands across samples. For the CA group, WFC was positively associated with affective discomfort (*r* = .43), emotional dissonance (*r* = .39) and customer verbal aggression (*r* = .20). Affective discomfort was positively related to emotional dissonance (*r* = .43) and customer verbal aggression (*r* = .21). However, for the SC group, WFC was positively associated with affective discomfort (*r* = .41), customer verbal aggression (*r* = .33) and emotional dissonance (*r* = .20). Affective discomfort was positively related to customer verbal aggression (*r* = .21) and emotional dissonance (*r* = .20).

Analysis of variance between the two samples showed some differences. The CA group perceived more WFC, *t*(949) = 5.50, *p* < .01, affective discomfort, *t*(947,104) = 2.74, *p* < .01, emotional dissonance, *t*(948,931) = 3.39, *p* < .01, and customer verbal aggression, *t*(949) = 7.93, *p* < .01, than the SC group.

**Figure 2:**
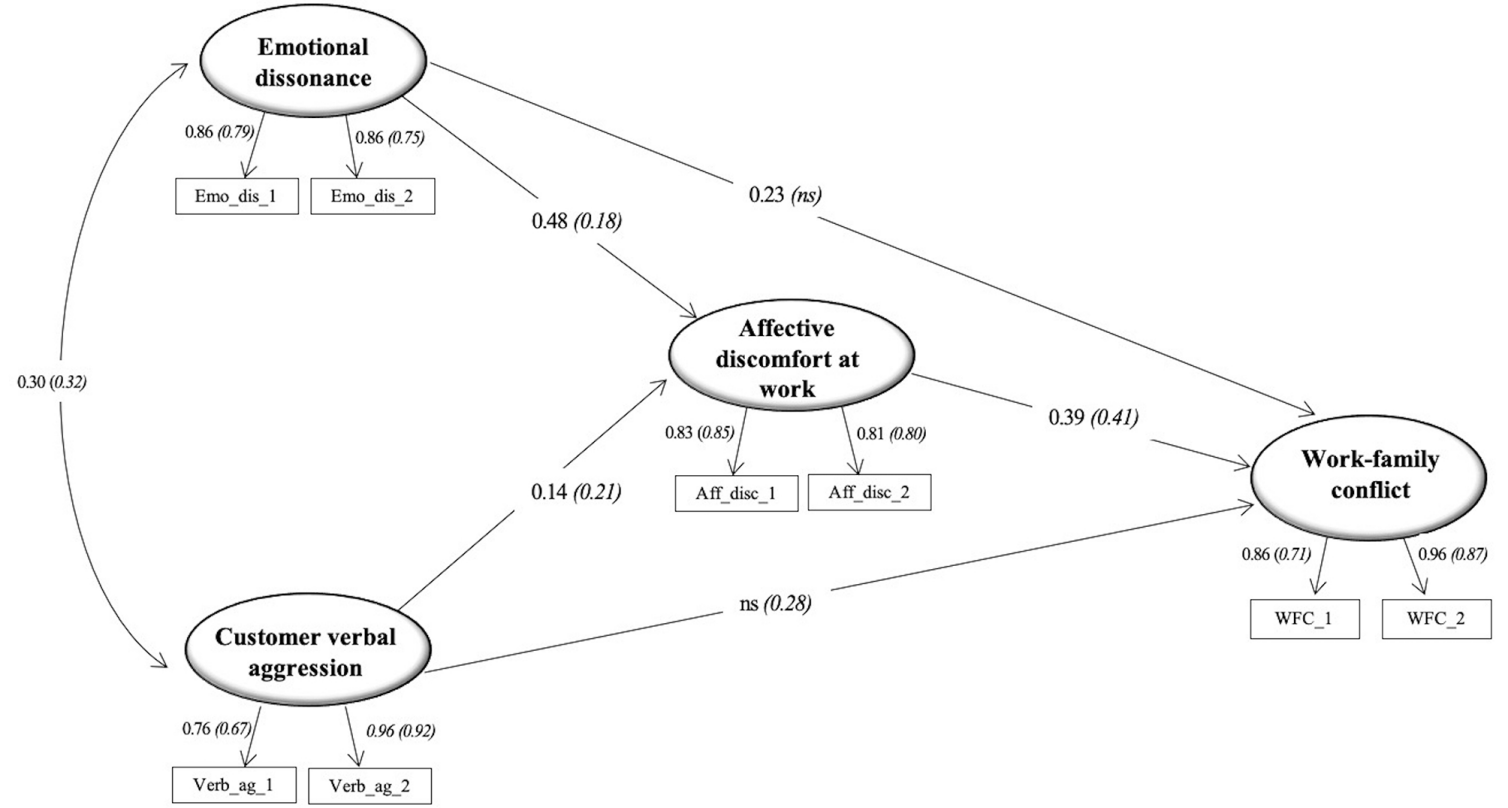
The model (standardised path coefficients, *p* < .01). *Note.* Results of the multi-group analysis: CA (SC). ns = non-significant relationship.

The multi-group SEM of the hypothesized model (Figure 1) was first evaluated by constraining all the path coefficients to be equal across the two groups. Subsequently, the model was re-tested relaxing the constraints that significantly increased the fit if they were estimated freely across the two groups, consistent with the theory (Bollen, 1989). The final model fitted to the data well: χ^2^ (37, *N*_CA_ = 507, *N*_SC_ = 444) = 136.26, *p* < .001, CFI = .97, TLI = .95, RMSEA = .07, 90% CI [.06, .09], SRMR = .04. A significant chi-square difference between the two models suggested this final model fitted the data better than the fully constrained model, Δχ^2^(4) = 21.80; *p* < .001 (Satorra & Bentler, 1999). Figure 2 shows the standardised parameters for both groups. By examining the estimated model, the variables showed good item loadings in both groups.

Emotional dissonance showed a positive direct relationship with WFC only in the CA group (CA: β = 0.23); customer verbal aggression was positively related to WFC only in the SC group (SC: β = 0.28). Emotional dissonance showed a significantly stronger positive relationship with affective discomfort in the CA sample than the SC sample (CA: β = 0.48; SC: β = 0.18). Customer verbal aggression was positively related to affective discomfort, across the two groups, stronger in the SC group (CA: β = 0.14; SC: β = 0.21). Finally, affective discomfort showed a significant positive relationship with WFC in both groups (CA: β = 0.39; SC: β = 0.41). Variance of dependent variables explained by the models was 29% for WFC and 29% for affective discomfort in the CA sample; 35% for WFC; and 10% for affective discomfort in the SC sample.

The estimated indirect effects are shown in Table 2 for the CA group and Table 3 for the SC group. In both groups, emotional dissonance and customer verbal aggression increased WFC through affective discomfort. More specifically, the model showed that in the CA sample the effect between customer verbal aggression and WFC was only indirect, mediated by affective discomfort; also affective discomfort mediated the relationship between emotional dissonance and WFC. In the SC group, the effect between emotional dissonance and WFC was only indirect, mediated by affective discomfort; also affective discomfort mediated the relationship between customer verbal aggression and WFC.

**Table 2:** Indirect Effects (N_CA_ = 507)

Indirect effect	Standardized indirect effect		
	Estimate	*SE*	*p*
Emo. Diss. → Aff. Disc. → WFC	.19	.03	.000
Cust. Verbal Aggr. → Aff. Disc. → WFC	.05	.02	.008

*Note.* Emo. Diss. = Emotional dissonance; Aff. Disc. = Affective discomfort at work; Cust. Verbal Aggr. = Customer verbal aggression; WFC = Work-family conflict.

**Table 3:** Indirect Effects (N_SC_ = 444)

Indirect effect	Standardized indirect effect		
	Estimate	*SE*	*p*
Emo. Diss. → Aff. Disc. → WFC	.07	.03	.007
Cust. Verbal Aggr. → Aff. Disc. → WFC	.09	.03	.001

*Note.* Emo. Diss. = Emotional dissonance; Aff. Disc. = Affective discomfort at work; Cust. Verbal Aggr. = Customer verbal aggression; WFC = Work-family conflict.

## Discussion

This study aimed to examine the relationship between job demands typical of service work (emotional dissonance and customer verbal aggression) and WFC, considering the mediational role of affective discomfort at work. Moreover, the study explored potential differences in these relationships between two professional groups, call centre agents and supermarket cashiers, characterised by service work, emotional labour and relation to customers.

The Hypotheses 1a and 1b stated that emotional dissonance and customer verbal aggression are positively associated with affective discomfort at work. Bivariate correlations and the estimated model showed a positive relation in both groups; emotional dissonance had a stronger relation with affective discomfort in the CA group whereas customer verbal aggression had a stronger relation in SC. Thus, H1 is confirmed. The findings suggested that emotional labour undertaken by service sector employees might have negative implications for their well-being and underlined the demanding role of these aspects (Schaufeli & Taris, 2014; Zapf, 2002). In fact, emotional dissonance was confirmed as a job demand associated with discomfort and detrimental consequences to employees’ health (Emanuel et al., 2014a; Hülsheger & Schewe, 2011; Kenworthy et al., 2014; Molino et al., 2016; Zapf et al., 1999). During voice-to-voice or face-to-face interactions, workers expressed emotions required by the organisations and met customer expectations and the relation is not always easy to manage (Kinman, 2009; Molino et al., 2016). Customer verbal aggression also confirmed its demanding and stressful role; aggression in working life might cause significant problems for an individual’s health and safety (Grandey et al., 2004; Judge et al., 2006). Customer-related stressor might be particularly taxing for frontline service workers who have frequent interactions with customers, but this relation could be problematic also for the organisation (Dormann & Zapf, 2004; Grandey et al., 2004). Frontline service providers are engaged in high levels of emotional labour and social interaction with customers and tended to experience more psychological strain as a consequence of workplace stress. Although employees in every sector are at risk of being exposed to aggressive behaviour, the risk is much greater for employees in the service sector, especially workers with a face-to-face relation and a physical proximity with customers, such as supermarket cashiers.

The Hypotheses 2a and 2b stated that emotional dissonance and customer verbal aggression are positively associated with WFC. Bivariate correlations showed a positive relation in both groups. The estimated model reported a positive association, with differences across groups: emotional dissonance had a direct effect on WFC only in the CA group while customer verbal aggression had a direct effect only in the SC group. H2 is therefore supported, with differences across groups. These results showed additional evidence that emotional dissonance is a work stressor is not merely related to negative consequences for psychological health and job outcomes, but is also a job demand that might affect the work-family interface (Cheung & Tang, 2009; Montgomery et al., 2005). According to previous studies, results confirmed the relationship between emotional labour and WFC (Carlson et al., 2012; Lawson et al., 2013; Montgomery et al., 2005; Sanz-Vergel et al., 2012; Seery et al., 2008; Wagner et al., 2014; Yanchus et al., 2010) and reported a difference between the two groups. This difference might be linked to the different relation with customers, voice-to-voice in CA and face-to-face with SC, and suggested that emotional labour executed by service sector employees was not a homogeneous experience (Kinman, 2009) but might be affected by demands, resources and organisational culture. Thus, it appears essentials conducting systematic investigations with different occupational groups, in order to investigate how different aspects of emotional labour might manifest themselves in the non-working domain.

Finally, the Hypothesis 3 stated that affective discomfort at work is positively related to WFC, thus playing a mediating role between emotional dissonance and customer verbal aggression on the one hand, and WFC on the other. Bivariate correlations and the estimated model confirmed a positive relation between affective discomfort and WFC in both groups, according to several studies reporting that emotions and affect from the workplace would transfer to the home and family domain (Grandey & Krannitz, 2016; Judge et al., 2006). The mediational role of affective discomfort at work among job demands and WFC presented differences among groups. In the CA sample, the indirect effect between customer verbal aggression and WFC mediated by affective discomfort was significant, whereas the direct effect was not significant. In the SC group, the indirect effect between emotional dissonance and WFC mediated by affective discomfort was significant, whereas the direct effect was not significant. These results suggested again that emotional labour executed by service sector employees is not a homogeneous experience (Kinman, 2009) and firstly supported the assumption that strain is likely to affect the perception and experience of WFC. Results underlined that high levels of job-related stressors and a lack of job resources might negatively affect employees’ well-being (Bakker & Demerouti, 2017) and WFC perception (Britt & Dawson, 2005; Ford et al., 2007; Kelloway et al., 1999; Kinnunen et al., 2004; Steinmetz et al., 2008; Westman et al., 2004).

### Limitations and Future Studies

The present study has several limitations that might have restricted the generalisability of our findings.

First, our study used a cross-sectional design that did not permit establishing causality relations among variables (Podsakoff, MacKenzie, & Podsakoff, 2012). Longitudinal studies are needed to determine the temporal relationship among these variables; in addition, future research could use other alternative methods, such as diary study, to collect information from participants.

A second limitation was the use of single-source self-report data, which raised questions about common method bias (Podsakoff et al., 2003). Future studies will use other data sources, including supervisors or colleagues’ data, observational data as well as objective measures, such as frequency, type and duration of customer interaction or physiological measures of occupational stress and well-being.

Third, the sample of service sector employees involved in this study is obtained from two different organisational contexts. The findings might not be representative of the wider population, as employees’ experiences might differ from other services organisations. Moreover, the employees delivered a service via the two different modalities (face-to-face and voice-to-voice) but do not perform identical tasks. Future research would benefit from examining the differences between two groups that perform identical job activities and differ only in the mode of customer contact. Furthermore, additional studies could also explore the relationship between variables and work perception, for example, in terms of perceived job insecurity, typical for customer service jobs in Italy, as done in other professional groups (e.g., Giunchi, Emanuel, Chambel, & Ghislieri, 2016).

Finally, this study considers only job demands, so in future research would be important to analyse the role of job resources and personal resources in the well-being and work-family interface dynamics, according to the JD-R theory (Bakker & Demerouti, 2017; Emanuel, Colombo, Gatti, Ghislieri, & Ricotta, 2011; Goussinsky, 2011; Schaufeli & Taris, 2014). This study provided some initial evidence that the impact of the type of delivery in emotion work is deserving of further investigation. Future research might adopt a qualitative approach to examine and better understand the differences in emotion work performed in face-to-face and voice-to-voice customer relations.

### Conclusion and Practical Implications

The results of the study contributed to the existing literature on WFC, affective discomfort at work and emotional labour in service occupations. Important implications for both researchers and practitioners emerged, in order to comprehend better which job demands, typical for customer service work, are more related to WFC and discomfort at work. Moreover, the study offered some initial suggestion to the possible impact of the type of delivery in emotion work and customer relations.

Furthermore, the results emphasised the importance of considering the characteristics of interaction with customers, recommending to differentiate organisational practices on the basis of relation to customers (face-to-face or voice-to-voice). In addition, it appeared fundamental to promote practices and interventions in order to support the management of emotions and the detrimental relationship with the customer in a targeted manner, for different occupations.

Several studies have shown that interventions to reduce WFC are relevant to promoting individual and organisational well-being (Amstad et al., 2011; Ghislieri & Colombo, 2014; Nohe et al., 2015). Organisations may offer formal work-family benefits; it is important that employees are aware of these benefits and the solutions should be addressed to workers’ expressed needs (Amstad et al., 2011). Moreover, organisations could foster work-family support from supervisors and co-workers, managers and supervisors should be sensitive and attentive to employees’ needs and provide necessary support to sustain a family-friendly climate at work (Ghislieri & Colombo, 2014).

The findings supported and extended previous research indicating that emotion labour is central to the role of front-line customer service employees and is related to a variety of strain outcomes. Moreover, stressors involving customers are an inevitable part of a frontline service job and could not be eliminated (Dormann & Zapf, 2004). In order to enhance employee well-being, it is necessary for organisations to recognise these risks, propose and implement interventions to help workers manage their emotions more effectively. Firstly, organisations could explain clearly to personnel the characteristics of emotional labour and the relationship with customers through a formal or written policy or procedure on how frontline employees could manage customer demands; these would convey a clear sense of the company’s expectations of its employees and customers.

Organisations and management could design and develop training programmes to enhance employees’ emotion regulation skills in order to cope with customer mistreatment (Grandey et al., 2004), reduce stress and sustain well-being and quality of life (Gabriel, Cheshin, Moran, & Van Kleef, 2016; Grandey & Krannitz, 2016). Training could help workers to comprehend better the detrimental consequences of emotional labour and identify appropriate strategies to cope job with demands. Training programmes might also help workers to manage customer interactions effectively, identify the most difficult situations, respond to customer requests and, at the same time, increase customer orientation and loyalty.

Referring to recruitment, it appeared important to make emotional requirements explicit during recruitment thus individuals could have a well-defined idea of what is expected and create expectations about emotional performance (Rafaeli & Sutton, 1987). Several scholars (Gabriel et al., 2016; Grandey & Gabriel, 2015) highlighted that competencies rooted in personality (positive attitude, sense of humour, enthusiasm, extraversion), technical skills (typing, navigation) and communication (energy, fluency, warmth, tone), should be used for selection in service jobs.

Finally, findings suggested the importance of monitoring the experiences of emotional dissonance and emotional labour and the negative consequences for employees, in order to sustain workers and promote well-being and a positive work-family balance in service jobs.

## Previously Presented

Some preliminary findings of this study were presented at the 19th EAWOP (European Association of Work and Organizational Psychology) Congress in Turin, Italy, June 2019.
